# Incidence of cleft lip and palate, and epidemiology of perinatal deaths related to cleft lip and palate in Hunan Province, China, 2016–2020

**DOI:** 10.1038/s41598-023-37436-y

**Published:** 2023-06-26

**Authors:** Xu Zhou, Yurong Jiang, Junqun Fang, Hua Wang, Donghua Xie, Haiyan Kuang, Ting Li, Qin Liu, Jian He

**Affiliations:** 1 Hunan Provincial Maternal and Child Health Care Hospital, Changsha, 410000 Hunan Province China; 2 The Hunan Children’s Hospital, Changsha, 410000 Hunan Province China; 3 National Health Commission Key Laboratory of Birth Defects Research, Prevention and Treatment, Hunan Provincial Maternal and Child Health Care Hospital, Changsha, 410000 Hunan Province China

**Keywords:** Medical research, Signs and symptoms

## Abstract

This study aimed to analyze the epidemiological characteristics of cleft lip and/or palate (CL/P) and CL/P-related perinatal deaths, provide some information for intervention programs to reduce the incidence of CL/P and provide clues for future researchers. Data were obtained from the Birth Defects Surveillance System in Hunan Province, China, 2016–2020. Incidences of CL/P [number of cases per 1000 fetuses (births and deaths at 28 weeks of gestation and beyond)] with 95% confidence intervals (CI) were calculated by residence, gender, maternal age, year, and major types [cleft lip only (CL), cleft palate only (CP), and cleft lip with palate (CLP)]. Crude odds ratios (ORs) were calculated to examine the association of each maternal characteristic with CL/P. Pearson chi-square tests (χ^2^) were used to examine the association of each maternal characteristic with CL/P-related perinatal deaths. A total of 847,755 fetuses were registered, and 14,459 birth defects were identified, including 685 CL/P (accounted for 4.74% of all birth defects). CL, CP, and CLP accounted for 24.67% (169 cases), 36.79% (252 cases), and 38.54% (264 cases) of all CL/P, respectively. The incidence of CL/P was 0.81‰ (95%CI 0.75–0.87). The incidence of CL was 0.20‰ (95%CI 0.17–0.23) (169 cases), of CP was 0.30‰ (95%CI 0.26–0.33) (252 cases), and of CLP was 0.31‰ (95%CI 0.27–0.35) (264 cases). CL was more common in males than females (0.24‰ vs. 0.15‰, OR = 1.62, 95%CI 1.18–2.22). CP was more common in urban than rural (0.36‰ vs. 0.25‰, OR = 1.43, 95%CI 1.12–1.83), and less common in males than females (0.22‰ vs. 0.38‰, OR = 0.59, 95%CI 0.46–0.75). CLP was more common in males than females (0.35‰ vs. 0.26‰, OR = 1.36, 95%CI 1.06–1.74). Compared to mothers 25–29 years old, mothers < 20 years old were risk factors for CLP (OR = 3.62, 95%CI 2.07–6.33) and CL/P (OR = 1.80, 95%CI 1.13–2.86), and mothers ≥ 35 years old was a risk factor for CLP (OR = 1.43, 95%CI 1.01–2.02). CL/P-related perinatal deaths accounted for 24.96% (171/685) of all CL/P, of which 90.64% (155/171) were terminations of pregnancy. Rural residents, low income, low maternal age, and early prenatal diagnosis are risk factors for perinatal death. In conclusion, we found that CP was more common in urban areas and females, CL and CLP were more common in males, and CL/P was more common in mothers < 20 or ≥ 35 years old. In addition, most CL/P-related perinatal deaths were terminations of pregnancy. CL/P-related perinatal deaths were more common in rural areas, and the proportion of CL/P-related perinatal deaths decreased with the increase in maternal age, parity, and per-capita annual income. Several mechanisms have been proposed to explain these phenomena. Our study is the first systematic research on CL/P and CL/P-related perinatal deaths based on birth defects surveillance. It is significant for intervention programs to prevent CL/P and CL/P-related perinatal deaths. As well, more epidemiological characteristics of CL/P (such as the location of CL/P) and approaches to reduce CL/P-related perinatal deaths need to be studied in the future.

## Introduction

Cleft lip and/or palate (CL/P) are classified as cleft lip only (CL), cleft palate only (CP), and cleft lip with palate (CLP)^1^. It significantly impacts life quality, healthcare use, and costs of patients and their families^2,3^ and increases the risk of perinatal deaths.

CL/P are common congenital malformations affecting the head and neck and one of the most common birth defects^4^. The global incidence of CL/P is reported to be 1.08‰^5^. Previous studies have shown Asia's highest incidence of CL/P^4,6–8^. China is one of the regions with the highest incidence of CL/P. E.g., a meta-analysis in China (1986–2015) showed that the overall incidence of orofacial clefts [referred to all types of clefts (CP, CL, or CLP; syndromic or nonsyndromic forms)] was 1.4 per 1000 live births^9^.

Through prenatal screening and diagnosis, doctors can diagnose CL/P early and reduce the incidence^10^. Therefore, studies on the epidemiological characteristics of CL/P are essential for providing evidence for future intervention. However, the epidemiology of CL/P and CL/P-related perinatal deaths have rarely been reported recently. More studies need to be included.

Therefore, we investigated the epidemiology of CL/P and CL/P-related perinatal deaths in Hunan Province, China, using data from the Birth Defects Surveillance System in Hunan Province, 2016–2020. This study aimed to provide some information for intervention programs to reduce the incidence of CL/P and to provide clues for future researchers.

## Methods

### Data sources

This study used data from the Birth Defects Surveillance System in Hunan Province, China, 2016–2020, which is run by the Hunan Provincial Health Commission and involves 52 representative registered hospitals in Hunan Province. Surveillance data of fetuses (births and deaths at 28 weeks of gestation and beyond) and all birth defects (between 28 weeks of gestation and 7 days after delivery) included demographic characteristics such as residence, gender, maternal age, and other key information.

According to the WHO International Classification of Diseases (Ninth Revision, ICD-9), the ICD code of CL is Q36, CP is Q35, and CLP is Q37.

### Informed consents

We confirmed that informed consent was obtained from all subjects and/or their legal guardian(s). Doctors obtain consent from pregnant women before collecting surveillance data, which is witnessed by their families and the heads of the obstetrics or neonatal departments. Doctors obtain consent from their parents or guardians for live births, which are witnessed by their families and the heads of the obstetrics or neonatal departments. Since the Health Commission of Hunan Province collects those data and the government has emphasized the privacy policy in the “Maternal and Child Health Monitoring Manual in Hunan Province”, there is no additional written informed consent.

### Ethics guideline statement

The Medical Ethics Committee of Hunan Provincial Maternal and Child Health Care Hospital approved the study (NO: 2022-S94). It is a retrospective study of medical records; all data were fully anonymized before we accessed them. Moreover, we de-identified the patient records before analysis. We confirmed that all experiments were performed following relevant guidelines and regulations.

### Data quality control

To carry out surveillance, the Hunan Provincial Health Commission formulated the “Maternal and Child Health Monitoring Manual in Hunan Province”. Data were collected and reported by experienced doctors. To reduce the integrity rate and information error rate, we asked the technical guidance departments to carry out comprehensive quality control each year.

### Statistical analysis

The incidence of CL/P is defined as the number of cases per 1000 fetuses (births and deaths at 28 weeks of gestation and beyond) (‰). We computed the incidences of CL/P (CL, CP, and CLP) and 95% confidence intervals (CI) by Poisson’s regression. Crude odds ratios (ORs) were calculated to examine the association of each maternal characteristic with CL/P. Chi-square trend tests (χ^2^_trend_) were used to determine trends in incidences of CL/P by year. Pearson Chi-Square tests (χ^2^) were used to examine the association of each maternal characteristic with CL/P-related perinatal deaths.

All statistical analyses in this study were performed using SPSS 18.0 (International Business Machines Corporation, New York City, United States).

## Results

### CL/P in Hunan Province, China, 2016–2020

Our study included 847,755 fetuses, and 14,459 birth defects were identified, including 685 CL/P (accounted for 4.74% of all birth defects). CL, CP, and CLP accounted for 24.67% (169 cases), 36.79% (252 cases), and 38.54% (264 cases) of all CL/P, respectively. Table 1 shows the details of CL/P by year.

**Table 1:** CL/P in Hunan Province, China, 2016–2020.

Year	Number of fetuses (n)	Number of birth defects	CL		CP		CLP		CL/P (total)	
			n	Proportion in CL/P (total) (%)	n	Proportion in CL/P (total) (%)	n	Proportion in CL/P (total) (%)	n	Proportion in total birth defects (%)
2016	170,688	3107	36	23.84	35	23.18	80	52.98	151	4.86
2017	196,316	3533	51	30.72	51	30.72	64	38.55	166	4.70
2018	177,762	2900	28	21.54	59	45.38	43	33.08	130	4.48
2019	164,840	2643	24	19.20	64	51.20	37	29.60	125	4.73
2020	138,149	2276	30	26.55	43	38.05	40	35.40	113	4.96
Total	847,755	14,459	169	24.67	252	36.79	264	38.54	685	4.74

*CL* cleft lip only, *CP* cleft palate only, *CLP* cleft lip with palate, *CL/P* cleft lip and/or palate.

### Incidences of CL/P by residence, gender, and maternal age

The incidence of CL/P was 0.81‰ (95%CI 0.75–0.87). The incidence of CL was 0.20‰ (95%CI 0.17–0.23) (169 cases), of CP was 0.30‰ (95%CI 0.26–0.33) (252 cases), and of CLP was 0.31‰ (95%CI 0.27–0.35) (264 cases).

CL was more common in males than females (0.24‰ vs. 0.15‰, OR = 1.62, 95%CI 1.18–2.22). CP was more common in urban than rural (0.36‰ vs. 0.25‰, OR = 1.43, 95%CI 1.12–1.83) and less common in males than females (0.22‰ vs. 0.38‰, OR = 0.59, 95%CI 0.46–0.75). CLP was more common in males than females (0.35‰ vs. 0.26‰, OR = 1.36, 95%CI 1.06–1.74). Compared to mothers 25–29 years old, mothers < 20 years old were a risk factor for CLP (OR = 3.62, 95%CI 2.07–6.33) and CL/P (OR = 1.80, 95%CI 1.13–2.86), and mothers ≥ 35 years old was a risk factor for CLP (OR = 1.43, 95%CI 1.01–2.02). Table 2 shows the detailed incidences and ORs of CL/P by residence, gender, and maternal age.

**Table 2:** Incidences of CL/P by residence, gender and maternal age.

Variables	Number of fetuses (n)	CL			CP			CLP			CL/P (total)		
		n	Incidence (‰, 95%CI)	OR (95%CI)	n	Incidence (‰, 95%CI)	OR (95%CI)	n	Incidence (‰, 95%CI)	OR (95%CI)	n	Incidence (‰, 95%CI)	OR (95%CI)
Residence													
Urban	342,178	67	0.20 (0.15,0.24)	0.97 (0.71–1.32)	124	0.36 (0.30,0.43)	1.43 (1.12–1.83)	94	0.27 (0.22,0.33)	0.82 (0.63–1.05)	285	0.83 (0.74,0.93)	1.05 (0.90–1.23)
Rural	505,577	102	0.20 (0.16,0.24)	Reference	128	0.25 (0.21,0.30)	Reference	170	0.34 (0.29,0.39)	Reference	400	0.79 (0.71,0.87)	Reference
Gender													
Male	448,288	109	0.24 (0.20,0.29)	1.62 (1.18–2.22)	100	0.22 (0.18,0.27)	0.59 (0.46–0.75)	159	0.35 (0.30,0.41)	1.36 (1.06–1.74)	368	0.82 (0.74,0.90)	1.04 (0.89–1.21)
Female	399,368	60	0.15 (0.11,0.19)	Reference	152	0.38 (0.32,0.44)	Reference	104	0.26 (0.21,0.31)	Reference	316	0.79 (0.70,0.88)	Reference
Unknown	99	0	–	–	0	–	–	1	–	–	1	–	–
Maternal age (years old)													
< 20	13,711	4	0.29 (0.07,0.74)	1.47 (0.54–4.02)	1	0.07 (0.01,0.41)	0.25 (0.03–1.80)	14	1.02 (0.56,1.71)	3.62 (2.07–6.33)	19	1.39 (0.84,2.16)	1.80 (1.13–2.86)
20–24	118,531	13	0.11 (0.06,0.19)	0.55 (0.31–1.00)	35	0.30 (0.21,0.41)	1.02 (0.69–1.49)	44	0.37 (0.27,0.50)	1.31 (0.92–1.87)	92	0.78 (0.62,0.93)	1.01 (0.79–1.27)
25–29	357,582	71	0.20 (0.15,0.24)	Reference	104	0.29 (0.23,0.35)	Reference	101	0.28 (0.23,0.34)	Reference	276	0.77 (0.68,0.86)	Reference
30–34	243,649	48	0.20 (0.14,0.26)	0.99 (0.69–1.43)	82	0.34 (0.26,0.41)	1.16 (0.87–1.55)	59	0.24 (0.18,0.30)	0.86 (0.62–1.18)	189	0.78 (0.67,0.89)	1.00 (0.84–1.21)
≥ 35	114,282	33	0.29 (0.20,0.41)	1.45 (0.96–2.20)	30	0.26 (0.18,0.37)	0.90 (0.60–1.35)	46	0.40 (0.29,0.54)	1.43 (1.01–2.02)	109	0.95 (0.77,1.13)	1.24 (0.99–1.54)
Total	847,755	169	0.20 (0.17,0.23)	–	252	0.30 (0.26,0.33)	–	264	0.31 (0.27,0.35)	–	685	0.81 (0.75,0.87)	–

*CL* cleft lip only, *CP* cleft palate only, *CLP* cleft lip with palate, *CL/P* cleft lip and/or palate, *CI* confidence intervals, *OR* crude odds ratio.

### Incidences of CL/P by year

From 2016 and 2020, the incidences of CL/P were 0.88‰ (95%CI 0.74, 1.03), 0.85‰ (95%CI: 0.72, 0.97), 0.73‰ (95%CI 0.61, 0.86), 0.76‰ (95%CI 0.63, 0.89), and 0.82‰ (95%CI 0.67, 0.97), respectively, and showed generally steady (χ^2^_trend_ = 1.19, *P* = 0.28). Table 3 shows the detailed incidences of CL/P by year.

**Table 3:** Incidences of CL/P by year.

Types	2016 (N: 170,688)		2017 (N: 196,316)		2018 (N: 177,762)		2019 (N: 164,840)		2020 (N: 138,149)		χ^2^_trend_	*P*
	n	Incidence (‰,95%CI)	n	Incidence (‰,95%CI)	n	Incidence (‰,95%CI)	n	Incidence (‰,95%CI)	n	Incidence (‰,95%CI)		
CL	36	0.21 (0.15,0.29)	51	0.26 (0.19, 0.33)	28	0.16 (0.10, 0.23)	24	0.15 (0.09, 0.22)	30	0.22 (0.15, 0.31)	1.23	0.27
CP	35	0.21 (0.14,0.29)	51	0.26 (0.19, 0.33)	59	0.33 (0.25, 0.42)	64	0.39 (0.29, 0.48)	43	0.31 (0.23, 0.42)	7.07	0.01
CLP	80	0.47 (0.37,0.57)	64	0.33 (0.25, 0.41)	43	0.24 (0.17, 0.33)	37	0.22 (0.16, 0.31)	40	0.29 (0.21, 0.39)	11.99	0.00
CL/P (total)	151	0.88 (0.74,1.03)	166	0.85 (0.72, 0.97)	130	0.73 (0.61, 0.86)	125	0.76 (0.63, 0.89)	113	0.82 (0.67, 0.97)	1.19	0.28

*CL* cleft lip only, *CP* cleft palate only, *CLP* cleft lip with palate, *CL/P* cleft lip and/or palate, *N* number of fetuses, *CI* confidence intervals.

### CL/P-related perinatal deaths

CL/P-related perinatal deaths accounted for 24.96% (171/685) of all CL/P, and 90.64% (155/171) of CL/P-related perinatal deaths were terminations of pregnancy. Perinatal deaths due to CL, CP, and CLP accounted for 8.88%, 3.97%, and 55.30% of all cases, respectively. And terminations of pregnancy due to CL, CP, and CLP accounted for 80.00%, 50.00%, and 94.52% of all deaths, respectively. The proportion of CLP-related perinatal deaths was relatively high. (χ^2^ = 212.38, *P* = 0.00). Table 4 shows the details of CL/P-related perinatal deaths.

**Table 4:** CL/P-related perinatal deaths.

Types	Number of cases (n)	Number of deaths (n)	Proportion of deaths in cases (%)	Number of termination of pregnancy (n)	Proportion of deaths due to terminations (%)	χ^2^	*P*
CL	169	15	8.88	12	80.00	212.38	0.00
CP	252	10	3.97	5	50.00		
CLP	264	146	55.30	138	94.52		
CL/P (total)	685	171	24.96	155	90.64		

*CL* cleft lip only, *CP* cleft palate only, *CLP* cleft lip with palate, *CL/P* cleft lip and/or palate.

### Epidemiology of CL/P-related perinatal deaths

Table 5 showed the following epidemiological characteristics of CL/P-related perinatal deaths: (1) CL/P-related perinatal deaths were more common in rural than urban areas. (2) The proportion of CL/P-related perinatal deaths decreased with the increase in maternal age, parity, and per-capita annual income. (3) The proportion of CL/P-related perinatal deaths was higher in the B-Ultrasound diagnosis group. And the earlier the diagnosis, the higher the proportion of CL/P-related perinatal deaths. Table 5 shows the detailed epidemiology of CL/P-related perinatal deaths.

**Table 5:** Epidemiology of CL/P-related perinatal deaths.

Characteristics	Number of CL/P (n)	Number of perinatal deaths (n)	Proportion of CL/P-related perinatal deaths (%)	χ^2^	*P*
Residence				14.34	0.00
Urban	285	50	17.54		
Rural	400	121	30.25		
Gender				4.35	0.11
Male	368	98	26.63		
Female	316	72	22.78		
Unknown	1	1	100.00		
Maternal age (years old)				18.90	0.00
< 20	19	10	52.63		
20–24	92	32	34.78		
25–29	276	73	26.45		
30–34	189	36	19.05		
≥ 35	109	20	18.35		
Maternal education				4.30	0.12
Secondary school or below	209	62	29.67		
Senior school	275	67	24.36		
University or above	201	42	20.90		
Per-capita annual income (￥)				12.49	0.00
< 4000	101	39	38.61		
4000–7999	194	48	24.74		
≥ 8000	390	84	21.54		
Parity				11.49	0.01
0	7	4	57.14		
1	302	89	29.47		
2	299	65	21.74		
≥ 3	77	13	16.88		
Diagnostic methods				283.22	0.00
B-Ultrasound	254	155	61.02		
Clinical	419	13	3.10		
Other	12	3	25.00		
Time of diagnosis				290.15	0.00
Antepartum (< 32 weeks)	173	111	64.16		
Antepartum (32–36 weeks)	73	42	57.53		
Antepartum (≥ 37 weeks)	20	5	25.00		
Postpartum (Within 7 days)	419	13	3.10		

*CL* cleft lip only, *CP* cleft palate only, *CLP* cleft lip with palate, *CL/P* cleft lip and/or palate.

## Discussion

Overall, we found a relatively high incidence of CL/P, and residence, gender, and maternal age impacted the incidence of CL/P. In addition, we found that CL/P-related perinatal deaths were associated with some epidemiological characteristics.

The incidence of CL/P (0.81‰) in our study is lower than the global incidence (1.08‰) or the reported incidence in China (1.4‰)^5,9^. Another comprehensive study by Cooper et al. also reported higher incidences of CL/P than ours: the incidences of CL/P in Chinese, Japanese, and other Asians were 1.30‰, 1.34‰ and 1.47‰, and the total incidence of CL/P in Asians was 1.33‰ (2006)^11^. The following are incidences of CL/P reported in some regions: 1.11‰ in South Korea, 2005–2006^12^, 1.5‰ in the Netherlands, 2008–2012^13^, 1.64‰ in the Czech Republic, 1994–2008^14^, 0.6‰ in Colombia, 2009–2017^15^, 0.65‰ in Saudi Arabia, 2013–2016^16^, and 0.76‰ in Guangdong Province, China, 2015–2018^17^. There are significant differences between them. And it seems the incidences of CL/P were higher in high-income countries. Several studies have also reported incidences of CL, CP, and CLP. E.g., the global incidences of CL, CP, and CLP were 0.3‰, 0.33‰, and 0.45‰, respectively^5^; The incidences of CL, CP, and CLP in South Korea were 0.28‰, 0.56‰ and 0.27‰ respectively^12^; The incidences of CL, CP, and CLP in Guangdong Province, China, were 0.23‰, 0.22‰ and 0.30‰, respectively^17^; The incidences of CL, CP, and CLP in Fangshan District, Beijing, China, were 0.62‰, 0.34‰ and 0.94‰, respectively^18^. There are also significant differences between them.

We infer that several factors may be related to these differences. First, it may reflect the frequency of defective genes for CP/L in different regions. E.g., Mitchell et al. found that the incidence of CL/P was higher in Asians than Caucasians than Africans^6^. Second, differences in access to diagnostic services may contribute to these results. Better access to diagnostic services for pregnant women in high-income countries resulted in more defects being detected^19^. The reason for the lower incidence of CL/P in this study may also be mainly related to prenatal screening and diagnosis (such as the use of B-ultrasound), and many fetuses with CL/P were diagnosed and terminated before 28 weeks of gestation. Third, some other factors may also be related to these differences. E.g., many studies mentioned above include relatively few cases or limited data.

We also found that CL/P was associated with residence, gender, and maternal age. E.g., CP was more common in urban than rural areas. It is consistent with some studies in low- and middle-income countries, such as Brazil and Jordan^20,21^, and inconsistent with some studies in high-income countries, such as the US and South Korea^22,23^. It may be mainly related to lower access to healthcare and diagnostic technologies for birth defects in rural areas^24^. In addition, some adverse conditions in urban areas may also contribute to this phenomenon, such as air pollution^25–28^. CL and CLP were more common in males, while CP was more common in females. It is consistent with previous studies^14,29–32^. The higher incidences of CL and CLP in males might be caused by the higher sensitivity of male fetuses to environmental stress, leading to the appearance of congenital birth defects^14,33^. CL/P was more common in mothers < 20 or ≥ 35 years old. It is consistent with previous studies^34–38^. It may be related to low-quality oocytes and semen for parents aged ≥ 35 years old^34,39–41^. And mothers < 20 years old may lack physical maturity and have a deficiency in vitamins (such as folic acid), which has been associated with birth defects^42^.

In addition, we found that CL/P-related perinatal deaths were associated with residence, maternal age, income, parity, methods, and time of diagnosis. It has been rarely reported recently. First, CL/P-related perinatal deaths were more common in rural areas, and the proportion of CL/P-related perinatal deaths decreased with the increase in maternal age, parity, and per-capita annual income. And more than 90% of CL/P-related perinatal deaths were terminations of pregnancy. It suggests that CL/P-related perinatal deaths may be mainly related to economic conditions^43–46^. CL/P significantly impacts babies’ life quality, healthcare use, and costs of patients and their families^7^. Therefore, better economic conditions benefit children's treatment and may make birth more likely. Second, CL/P-related perinatal deaths were associated with the methods and time of diagnosis. It is mainly concerned with the health of mothers. On the one hand, most CL/P-related perinatal deaths are therapeutic terminations of pregnancy^4,47^. On the other hand, the earlier CL/P is diagnosed and terminated, the less adverse impact termination has on mothers and their families. Therefore, most mothers terminate as soon as possible after diagnosing CL/P. Currently, B-Ultrasound is the most common and effective prenatal screening and diagnostic method, and many CL/P can be detected early in pregnancy by B-Ultrasound. Therefore, the proportion of CL/P-related perinatal deaths was higher in the B-Ultrasound diagnosis group.

Some things could be improved in our study. E.g., we did not analyze some epidemiological features due to data limitations, including the father’s information and the location of CL/P (left, center, or right). To address these limitations, first, the Monitoring Manual for Birth Defects Surveillance needs improvement; second, more studies should focus on the epidemiological characteristics of CL/P.

## Conclusion

In conclusion, we found that CP was more common in urban areas and females, CL and CLP were more common in males, and CL/P was more common in mothers < 20 or ≥ 35 years old. In addition, most CL/P-related perinatal deaths were terminations of pregnancy. CL/P-related perinatal deaths were more common in rural areas, and the proportion of CL/P-related perinatal deaths decreased with the increase in maternal age, parity, and per-capita annual income. Several mechanisms have been proposed to explain these phenomena. Our study is the first systematic research on CL/P and CL/P-related perinatal deaths based on birth defects surveillance. It is significant for intervention programs to prevent CL/P and CL/P-related perinatal deaths. As well, more epidemiological characteristics of CL/P (such as the location of CL/P) and approaches to reduce CL/P-related perinatal deaths need to be studied in the future.

## Data Availability

All data generated or analysed during this study are included in this published article.
